# HIV-1 persistent viremia is frequently followed by episodes of low-level viremia

**DOI:** 10.1007/s00430-017-0494-1

**Published:** 2017-02-20

**Authors:** Marek Widera, Miriam Dirks, Barbara Bleekmann, Robert Jablonka, Martin Däumer, Hauke Walter, Robert Ehret, Jens Verheyen, Stefan Esser

**Affiliations:** 1 Institute of Virology, University Hospital, University of Duisburg-Essen, Virchowstr. 179, 45147 Essen, Germany; 2 Clinic of Dermatology, University Hospital, University of Duisburg-Essen, Essen, Germany; 3 Institut für Immunologie und Genetik, Kaiserslautern, Germany; 4 Laboratory MIB, Medical Infectiology Center Berlin, Berlin, Germany

**Keywords:** HIV-1, Persistent viremia (PV), Low-level viremia (LLV), Drug resistance, Prolonged decline, Antiretroviral therapy

## Abstract

After the start of antiretroviral therapy (ART), plasma HIV-RNA levels should fall below the limit of detection (LOD) within 24 weeks. Hence, the prolonged decline of HIV-RNA after ART initiation is defined as persistent viremia (PV). In this retrospective study, we analyzed factors associated with PV. Next-generation sequencing of viral RNA/DNA was performed to study viral evolution and the emergence of drug-resistance mutations in HIV-infected patients with PV (*n* = 20). In addition, HIV-DNA species, immunological parameters, and clinical data of the patients were analyzed. We found that the possible causes for PV were divers, and both virologic and host parameters of this particular cohort were heterogeneous. We identified viruses with therapy-associated DRMs in six patients (30%); two of these were detected as minority variants. Five patients had sub-optimal drug levels (25%) and the baseline plasma viral loads were relatively high. Strikingly, we observed that >40% of the PV patients finally reaching HIV levels below the LOD later on showed up with episodes of low-level viremia (LLV). However, the amount of PBMC derived HIV-DNA species was not correlated with the likelihood of LLV after PV. According to our data, we conclude that drug-resistant viruses, sub-optimal drug level, and high baseline viral loads might be probable reasons for the prolonged RNA decline only in a sub-set of patients. In the absence of emerging DRMs and/or compliance issues, the clinical implications of PV remain unclear; however, PV appears to be a risk factor for episodes of LLV.

## Introduction

After the start of antiretroviral therapy (ART), HIV-RNA levels decline in a multiphasic manner. After a rapid drop of plasma viral RNA levels in the blood within days, a second phase of slower RNA decline below the clinical detection limit (<50 copies/ml) is observed [1, 2]. Even after several years of effective ART, a residual viremia can be detected with ultrasensitive methods. The reasons for this may be the virus release from reservoirs or remaining viral replication [3, 4]. In clinical routine, the goal of antiretroviral therapy is to suppress the viral load to undetectable levels, which, in clinical practice, is usually achieved within 6 months [2, 5]. Since the rate of viral decay after ART initiation is related to the half-life of the virus producing cells [6], a prolonged decline might be associated with the presence of long-lived productively infected cells like macrophages and latently infected CD4^+^ T cells, which can be stimulated to produce virus upon interaction with specific antigens [1]. Furthermore, cells present in sanctuary sites, which are difficult to target by the immune response and by drugs, were shown to contribute to the HIV-1 RNA decline [1, 2, 7–9]. Recently, it has been shown that persistent HIV-1 replication can maintain in tissue reservoirs during therapy and that HIV-1 can continue to replicate and refill those viral reservoirs despite effective ART [8]. Thus, the persistence of virus in cellular reservoirs can negatively influence effective suppression [10, 11].

In addition, strict compliance with the ART is a crucial precondition to sustainable control of HIV viral load and to low risk of developing drug resistance. Here, sub-optimal ART adherence was associated with increased residual virus quantity; however, residual viremia can also occur in complete ART adherence [3, 12]. Furthermore, malabsorption, insufficient dosage, drug–drug interactions, impaired intracellular metabolism, P450 hyperactivity, and P glycoprotein overexpression might have a tremendous effect on ART efficacy [13].

In a recent study, it has been shown that patients having a high pre-ART pVL show a poor probability of achieving viral suppression when compared to patients with lower BL pVL [4, 14]. In particular, patients with >500,000 copies/ml showed a low probability to achieve viral suppression within 72 weeks [15].

The emergence of drug-resistance mutations (DRMs) is one of the major causes leading to therapy failure. Here, pre-existing drug-resistant HIV strains can also have dramatic impact on ART efficacy. In particular, transmitted variants carrying DRMs are found in approx. 10% (12.6% in USA and 8.8% in Europe) of treatment-naïve (TN) patients [16, 17]. In addition, the high-error rate of the virus encoded reverse transcriptase (RT) might also facilitate the early selection of drug-resistant HIV already present at ART baseline as minority variant in the pool of quasi species [18–20]. Particularly, patients with prolonged viral decline were shown to have a high risk of selecting drug-resistant viruses [21].

So far, the underlying mechanism of PV has, however, remained elusive. Thus, in this study, we analyzed patients with persistent HIV viremia and studied the clinical patterns of PV, the viral evolution of DRMs using next-generation sequencing (NGS), and the long-term outcome after finally reaching plasma viral load (pVL) under the limit of detection (LOD).

## Materials and methods

### Study design

As a part of a retrospective analysis of HIV-1 positive patients treated with antiretroviral therapy (ART) in the HIV outpatient center at the University Hospital Essen (Germany) from 2013 to 2016, we studied 20 individuals who had pVL > 50 copies/ml for longer than 24 weeks after the start or restart of antiretroviral treatment (Table 1; Fig. 3). This study has been approved by the Ethics Committee of the medical faculty of the University Duisburg-Essen (14-6155-BO) and has been registered with the ClinicalTrials.gov database under No. NCT02411071.

**Table 1:** Participant characteristics

*n* = 20	Baseline (Pre-ART), median (IQR)	Genotyping (during ART) median (IQR)
HIV-1 RNA (cp/ml)	302,000 (39,473; 744,150)	136 (76; 300)
Immunological parameters		
CD4+ T cells/µl	105.5 (55.5; 286.8)	294 (234; 364)
HLA-DR+ T cells/µl	387 (177; 591)	301 (128; 473)
Ratio CD4/CD8	0.21 (0.095; 0.42)	0.56 (0.3; 0.56)
Antiretroviral treatment (ART)		
Duration		
BL → GT (days)	237 (172; 318)	
BL → LOD (days)	459 (342; 649)	
Regimens (%)		
DRV/r + 2 NRTI	12	
ATV/r + 2 NRTI	1	
LPV/r + 2 NRTI	2	
EFV + 2 NRTI	2	
RAL/EVG + 2 NRTI	2	
DRV/r + DTG	1	

Patient parameters were analyzed before ART initiation (base line = BL) and during persistent viremia at the time point of genotyping (GT). Flow cytometric determined CD4+ T-cell levels were obtained from routine diagnostics

*ATV* Atazanavir, *DRV* Darunavir, *LPV* Lopinavir, *EFV* Efavirenz, *RAL* Raltegravir, *EVG* Elvitegravir, *DTG* Dolutegravir, */r* boosted with Ritonavir, *IQR* interquartile range, *BL* Baseline, *GT* genotyping, *LOD* limit of detection (<50 HIV-1 RNA cp/ml)

### HIV quantification and genotyping analysis

Plasma HIV-RNAs were quantified using the Abbott RealTime HIV-1 m2000 test system as described by the manufacturer. Viral RNA and the corresponding proviral DNA of the 20 EDTA samples were isolated, amplified, sequenced [protease (PR) and reverse transcriptase (RT)], and analyzed for the presence of drug-resistance mutations (DRMs). Here, HIV-RNA and DNA were isolated using QIAamp Viral RNA Kit (Qiagen) and QIAamp DNA Blood Mini Kit (Qiagen), respectively, according to the manufacturer’s instructions. RNA was reverse transcribed using one-step RT-PCR kit (Qiagen), and PR and RT sequences were amplified using the Hot-StarTaq polymerase kit (Qiagen) according to a nested PCR protocol published previously [22]. Accordingly, DNA was subjected to the same nested PCR procedure. The genetic diversity of each sample was analyzed by next-generation sequencing (NGS) using the Illumina MiSeq platform during ART, which was compared to HIV drug-resistance tests previously performed in routine diagnostics (Sanger Sequencing). DRMs as listed by the International AIDS Society (IAS) were scored as major variants when detected in more than 10% of the reads and as minority variants when detected in less than 10%. As far as possible, the resistance testing of samples with low viral load was performed from RNA (*n* = 16); otherwise, proviral DNA was sequenced (*n* = 20).

### Measurements of HIV total, integrated proviral, and episomal 2-LTR circle DNA

Total and integrated proviral as well as two long terminal repeat (2-LTR) circle HIV-DNA were quantified using a pre-PCR followed by a real time PCR as described previously [23]. Briefly, peripheral blood mononuclear cells (PBMCs) were extracted by Ficoll centrifugation using LEUCOSEP tubes with porous barrier (grainer bio-one) and the DNA was isolated using QIAamp DNA Blood Mini Kit (Qiagen) according to the manufacturer’s instructions. To maintain exponential amplification, 12 cycles of HIV-DNA and the CD3 gene preamplification were carried out using thermal cycler T professional Trio (Biometra). The pre-PCR products were diluted and used as templates for the second round of amplification, which was performed on a Rotor-Gene-Q instrument (Qiagen). Standard curves were created with plasmid DNA templates harboring the amplicon sequences. A plasmid-containing nucleotide sequences from the human CD3gamma and HIV-1 pNL4-3 [24] derived 2-LTR circles (pEX-K4-2-LTR-CD3) was synthesized and purchased by Eurofins Scientific. For total HIV determination, the pre-PCR amplicon was cloned using PCR cloning kit (NEB) resulting in the plasmid pMiniT NL4-3 LTR-In1. For the generation of the PCR standard for quantitative Alu PCRs, genomic DNA from LTR-containing TZM-bl cells was isolated using QIAamp DNA Blood Mini Kit (Qiagen) according to the manufacturer’s instructions and copy-corrected by calculation of the LTR/CD3 ratio. All PCR standards were prepared by serial tenfold dilutions.

### Liquid chromatography–mass spectrometry for drug-level analysis

Following a protein precipitation, drug levels were determined by an in house high-performance liquid chromatography–mass spectrometry (HPLC/MS/MS). Quantification was performed on the basis of a dilution series used on each run. Each controls for low and high are based on weighing of pure substance. For evaluation of the drug efficacy, the trough levels (*C*_min_) of each drug were correlated to the corresponding therapy application. The *C*_min_ and *C*_max_ values are obtained from the average measured values after intake in healthy volunteers as described in the corresponding patient information leaflet provided by the manufacturer. For darunavir, there are two *C*_min_ and *C*_max_ values depending on the mode of ingestion.

### Statistical analysis

The statistical significance of the different decline periods between ART initiation and first pVL <50 copies/ml of patients with and without treatment switch during PV was estimated by performing a two-tailed Mann–Whitney test. Significance of correlation was calculated using two-tailed Pearson correlation coefficient. *P* values <0.05 were evaluated as significant.

## Results

### Patient characteristics

In our study, 15 patients received a first-line therapy (FL), while five of them were treatment experienced and restarted ART after treatment interruption. Before ART initiation, the median pVL was 302,000 copies/ml and dropped to 136 copies/ml during 237 days (IQR 172; 318) with ART at the time of genotyping. The overall pVL decline after the ART initiation (if has been successful) required 459 days (IQR 342; 649) to reach pVL <50 copies/ml (*n* = 17). Three patients did not reach pVL below the LOD and were lost to follow up. 16 of the 20 patients were treated with a PI-containing regimen combined with a NRTI-backbone, whereas only few of them received treatment regimens based on integrase inhibitors (INSTI) or NNRTI (Tables 1, 2). As determined by Sanger sequencing of proviral DNA, only in a single patient (PV-04), HIV carrying the RT associated M184V drug-resistant mutation (DRM) was detected in routine diagnostics prior to the therapy switch. Overall, patients in this analysis cohort had relatively low CD4 counts and high plasma HIV-1 RNA levels. As shown in Table 1, median baseline HIV-1 RNA level (pre-ART) was 302,000 (39,473; 744,150) copies/ml. Median baseline (BL) CD4+ T cells level was 105.5 cells/µl (55.5; 286.8) with a CD4/CD8 ratio of 0.21 (0.095; 0.42). CD4+ levels as measured at times genotyping (GT) was 294 cells/µl (234; 364) with a CD4/CD8 ratio of 0.56 (0.3; 0.56). The pre-ART HLA-DR+ T-cell level was 387 cells/µl (177; 591) and dropped to 301 cells/µl (128; 473) during ART. Samples were obtained between the 26th and 52nd week after ART start or restart. Only in three therapy-experienced (TE) patients (PV-18, PV-19, and PV-20), samples were collected at a later time after ART restart, which were between the 65th and 117th week.

**Table 2:** Patient parameters before ART initiation and during genotyping

ID	HIV-RNA pVL				Antiretroviral treatment					Course of viral load after PV					Resistance-associated mutations (PR /RT)	
	GT	BL														
	pVL	<100.000	>100.000	>500.000	FL/TE		ART during GT	New ART		Controlled pVL	Blips	LLV	All >50 long-term negative	All >50 with any event	DRMs in RNA	DRMs in DNA
PV 01	77			X	FL		ABC, 3TC, DRV/r → ABC, 3TC, RAL			<50		X		X	nd	No DRMs
PV 02	75		X		FL		TDF, FTC, DRV/r → FTC, TDF, ATV/r			<50		X		X	No DRMs	No DRMs
PV 03	310		X		FL		TDF, FTC, ATV/r → FTC, TDF, EVG			<50		X		X	I47IV (1%), H69K, L89M −	**M46IM (5%)**, I50IV (1%)
PV 04	205	X			FL		TDF, FTC, DRV/r → RAL, DRV/r			<50		X		X	Q58E Y181YC (1%)	Q58EQ, I62IV **M184V**
PV 05	109			X	FL		TDF, FTC, ***RAL*** → TDF, FTC, ATV/r			<50		X		X	No DRMs	No DRMs
PV 06	119			X	FL		TDF, FTC, ***EVG*** → TDF, FTC, DRV/r			<50			X		No DRMs	No DRMs
PV 07	89			X	FL		TDF, FTC, EFV → TDF, FTC, DTG			<50			X		– E138A, **G190A**	– E138A, **G190A**
PV 08*	366				FL		TDF, FTC, DRV/r → TDF, FTC, ***RAL***			<50			X		– K101EK (2%)	No DRMs
PV 09*	54		X		FL		TDF, FTC, DRV/r → TDF, FTC, RPV			<50			X		E138AE	L90LM
PV 10	47		X		FL		TDF, FTC, DRV/r			<50	X			X	– *K103N*, **M184V**	– *K103N*
PV 11	54		X		FL		TDF, FTC, EFV			<50			X		No DRMs	No DRMs
PV 12	643	X			FL		TDF, FTC, DRV/r			<50			X		No DRMs	No DRMs
PV 13	80	X			FL		TDF, FTC, DRV/r			<50	X			X	nd	No DRMs
PV 14	150		X		FL		TDF, FTC, DRV/r			<50		X		X	No DRMs	No DRMs
PV 15*	347			X	FL		TDF, FTC, LPV/r			Not <50					nd	no DRMs
PV 16	270			X	TE		TDF, FTC, LPV/r → TDF, FTC DRV			<50	X			X	**V82AV (2%)**, *N88DN (1%)* *D67DN*	*D30N (1%)* –
PV 17*	797			X	TE		TDF, FTC, DRV/r			Not <50					nd	No DRMs
PV 18*	179	X			TE		DRV/r, (RAL) → DRV/r, ***DTG***			Not <50					– *D67DN, M184V*	– *V108IV (2%), M184V*
PV 19	43		X		TE		TDF, FTC, DRV/r			<50			X		No DRMs	– **M184MV**
PV 20	183		X		TE		ABC, 3TC, DRV/r			<50			X		No DRMs	– *Y188CY (5%), E138EK*

ID	Drug level	Proviral DNA [/10^6^ cells]			Time periods						Immunological parameters					
											GT			Δ(BL- GT)		
		Total proviral DNA	Integrated DNA	2-LTR circles	BL—<50 cp/ml	GT—<50 cp/ml			GT—TS	TS—<50	CD4+ T cells [/µl]	HLA-DR+ T cells [/µl]	Ratio CD4/CD8	ΔCD4+ T cells (/µl)	ΔHLA-DR+ T cells (/µl)	ΔRatio CD4/CD8
PV 01		nd	nd	0.01	51	38			13	175	417	280	0.53	342	113	0.20
PV 02	DRV ↓	11901.0	521.9	0.1	55	9			3	42	188	68	0.51	166	36	0.33
PV 03		23.3	1319.3	154.8	187	102			10	645	234	401	0.33	19	−1689	0.25
PV 04		6772.3	521	0.1	82	35			4	216	294	121	0.70	206	82	0.42
PV 05		nd	353.7	3.6	72	32			11	146	330	322	0.34	255	−92	0.23
PV 06		0.4	1.2	nd	57	23			0	164	609	109	2.23	238	−162	1.32
PV 07		16373.0	1837.5	nd	755	50			0	351	44	98	0.37	33	12	0.28
PV 08*		80605.8	512.0	97.9	101	73			42	214	364	606	0.33	267	−391	0.26
PV 09*		11557.4	514	54.1	130	88			22	462	338	273	0.37	224	−537	0.25
PV 10		151.2	14.8	797.6	47	24					246	360	0.31	111	26	0.03
PV 11		2959.5	1158.9	29.1	56	22					432	369	0.75	73	−54	0.15
PV 12		132.3	2.8	nd	45	16					160	150	0.26	112	−222	0.19
PV 13	no TDF, FTC, DRV ↓	nd	nd	nd	24	3					448	221	0.56	123	−338	0.20
PV 14		12.3	1.7	nd	66	24					236	509	0.28	178	−50	0.21
PV 15*	nd	9624.3	112.6	0.2							270	275	0.18	257	89	0.14
PV 16	LPV ↓	36300.9	1944.8	nd	46	17			11	38	301	155	0.65	−27	−77	0.23
PV 17*	No TDF	nd	215.8	nd							125	70	0.47	−125	−193	−0.20
PV 18*	DTG ↓	27609.8	337.2	12.4							267	378	0.29	−6	128	0.00
PV 19		0.8	nd	nd	87	6					359	737	0.28	232	115	0.13
PV 20		14095.4	332.8	nd	99	26					474	820	0.47	89	430	−0.05

HIV viral loads before ART initiation and during GT were determined as described in the method section. In addition, therapy switches (→) of treatment experienced patients (TE) or patients with the first-line therapy (FL) regimen are given. The course of viral loads after PV describes whether VL after PV dropped below 50 copies/ml or other events like viremias, blips, or low-level viremia occurred as indicated with a cross. The previous drug-resistance mutations (DRMs) were obtained with Sanger sequencing. New DRMs were determined using next-generation sequencing (NGS). On the latter issue, mutations have been estimated in the indicated proportion of the reads (10, 5, and 1%). The sensitivity cutoff at 10% was assumed to be around the cutoff of Sanger sequencing. Therapy-associated drug resistance is marked in bold; ancient therapy-associated or primary DRMs are indicated in italic. INI are highlighted in bolditalic. Lost to follow-up patients is marked with an *. Drug levels were determined using LC-MS. (*nd* not detected; * lost to follow-up; bolditalic: intregrase inhibitors; bold: therapy-associated DRMs; italic: ancient therapy-associated or primary DRMs)

### DRMs detected in PV patients

To investigate whether a prolonged HIV-RNA decline might be correlated with the emergence of DRMs, we used next-generation sequencing (NGS) and sequenced plasma RNA as well as proviral DNA derived PCR amplicons. We identified viruses with therapy-associated DRMs in six patients (30%). As shown in Table 2, M184V was detected in the DNA derived from PV-04, which was already present before ART switching (see above). In patient PV-07, we identified G190A in both RNA and DNA derived samples indicating resistance to EFV, which was used as part of the first-line therapy. In patient PV-10, we detected the major NRTI mutation M184V in RNA indicating a resistance to FTC as part of the current ART regimen. Analyzing proviral DNA isolated from patient PV-19, we also detected a further mutation M184V. We were able to determine PI-associated DRMs in patient PV-16 in the minorities of the reads obtained from RNA (V82A; 2%). Furthermore, we identified M46I as a minority variant in 5% of proviral DNA in patient PV-03. In four patients (20%), we found DRMs, which were not related to the current therapy regiment but might be related to transmitted DRMs (FL, PV-10) or associated with previously used therapy regimens (TE, PV-16; PV-18; and PV-20). Since DRMs were only found in 25% of the analyzed RNA and DNA samples obtained from PV patients DRMs seem not to be considered as the only reason of PV in our cohort.

### Drug-level measurement in PV patients

Since reduced drug absorption might be another cause for PV, we analyzed the drug-level concentrations in each patients plasma at the time of genotyping. In most cases, drug levels were found to be in the therapeutic range (Tables 2, 3). However, in 25% of the patients (*n* = 5; PV-02, PV-13, PV-16, PV-17, and PV-18), we found that the concentration of at least one drug of the current ART regime was below the therapeutic drug level *C*_min_. In one of these cases, also a new DRM was detected during PV (PV-16: V82A). Hence, sub-optimal drug levels can also be a reason for PV in a sub-set of patients.

**Table 3:** LC-MS drug-level quantification of patient with persistent viremia at time point of genotyping

ID	ART	NNRTI	NRTI		PI						INI		
		EFV	TDF	FTC	ATV	DRV 2*600	DRV 1*800	LPV	cobi	rtv	RAL	EVG	DTG
PV-01	ABC, 3TC, DRV/r	–	–	–	–	–	6370	–	–	1020	–	–	–
PV-02	FTC, TDF, DRV/r	–	224	532	–	**2100**	–	–	–	1060	–	–	–
PV-03	TDF, FTC, ATV/r	–	65.4	798	2900	–	–	–	–	1430	–	–	–
PV-04	TDF, FTC, DRV/r	–	148	259	–	–	2580	–	–	185	–	–	–
PV-05	TDF, FTC, RAL	–	94.4	1460	–	–	–	–	–	–	2600	–	–
PV-06	TDF, FTC, EVG	–	279	1140	–	–	–	–	1810	–	–	1960	–
PV-07	TDF, FTV, EFV	1070	65.4	201	–	–	–	–	–	–	–	–	–
PV-08	TDF, FTC, DRV/r	–	63.8	203	–	–	4470	–	–	150	–	–	–
PV-09	TDF, FTC, DRV/r	–	63.2	203	–	–	1980	–	–	286	–	–	–
PV-10	FTC, TDF, DRV/r	–	271	890	–	–	6440	–	–	159	–	–	–
PV-11	TDF, FTC, EFV	1260	75.1	202	–	–	–	–	–	–	–	–	–
PV-12	TDF, FTC, DRV/r	–	127	1910	–	–	7360	–	–	513	–	–	–
PV-13	TDF, FTC, DRV/r	–	–	–	–	–	**687**	–	–	–	–	–	–
PV-14	TDF, FTC, DRV/r	–	191	1220	–	–	3750	–	–	308	–	–	–
PV-16	FTC, TDF, LPV/r	–	52.2	66.7	–	–	–	**257**	–	**25.2**	–	–	–
PV-17	TDF, FTC, DRV/r	–	–	69.9	–	–	986	–	–	**14.7**	–	–	–
PV-18	DRV/r, DTG	–	–	–	–	–	1270	–	–	67,6	–	–	**811**
PV-19	TDF, FTC, DRV/r	–	78.5	215	–	–	1950	–	–	151	–	–	–
PV-20	ABC, 3TC, DRV/r	–	–	–	–	–	5320	–	–	578	–	–	–
	Control_1	3820	2500	1540	1500	1550	1550	3970	1510	2120	1670	1190	1950
	Control_2	833	552	355	345	375	375	945	381	438	371	273	442
	*C*_min_	750	20	20	157	2430	841	1500	38	40	70	190	1110
	*C*_max_	5250	2500	2500	6340	6500	6060	14,000	1500	1230	2200	2090	4150

Drug levels at time point of genotyping were quantified using HPLC/MS/MS as described in the method section. In addition, two internal controls (Control_1; Control_2) were used as references. Bold values indicate too low concentration of the indicated drug (<*C*_min_). DRV 1*800 indicates a dosing scheme of 800 mg darunavir once-daily, DRV2*600 refers to the dosing of 600 mg twice-daily. The *C*_min_ and *C*_max_ values are obtained from the patient information leaflets provided by the manufacturer. Regardless of the mode of ingestion, values for darunavir were available for twice-daily (DRV 2*600) only. All values are given in ng/ml

### Impact on BL pVL and therapy switching on RNA decline duration

The duration of PV has been correlated with pVL before ART initiation where patients with high BL pVL showed a longer decline period when compared to those with low BL pVL [4, 14]. Furthermore, patients having a high BL pVL prior to ART begin (>500,000 copies/mL) were shown to have a poor probability to achieve full viral suppression [15]. Since most of the patients in our cohort had pVL >100,000 copies/ml (~80%, Table 2), which is defined as very high, our data suggest that high virus load levels might be considered as another reason for the prolonged virus decay. Because the patients analyzed in this study were already selected on the basis of the prolonged decline period, we did not observe significant different RNA decline durations within sub-groups (data not shown).

**Fig. 1:**
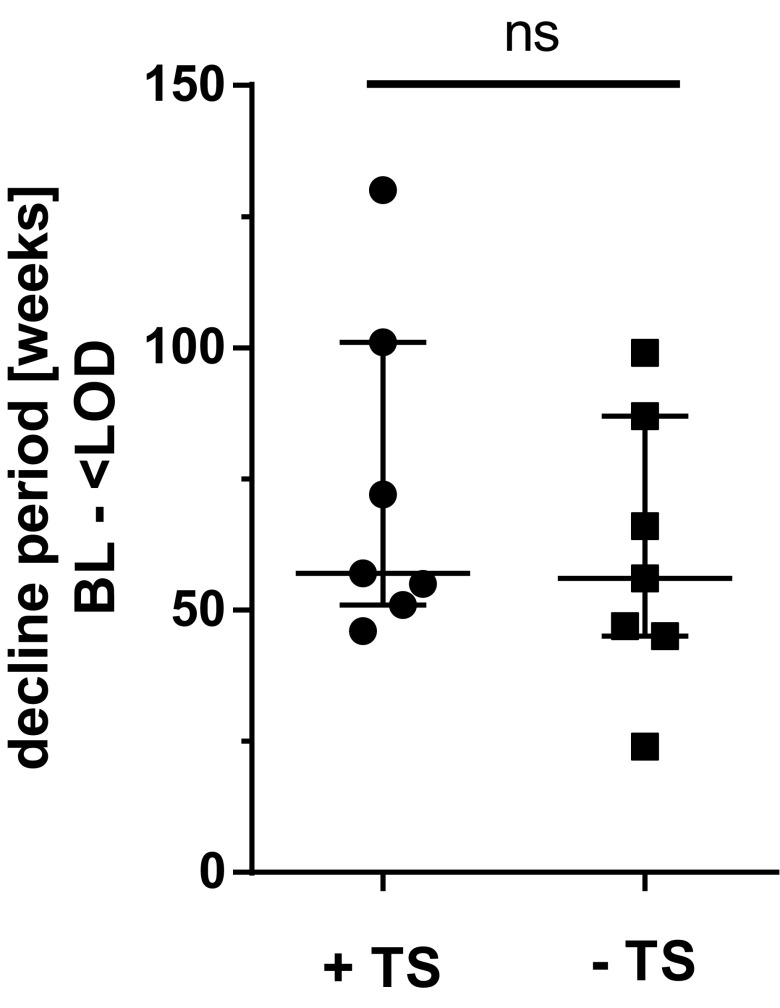
Duration of PV in patients with and without therapy switch: Median (IQR) of decline periods between genotyping and first pVL < LOD of patients with (+TS) and without treatment switch (−TS) after genotyping. Patients who were switched due to DRMs were excluded from this analysis. Significance was tested using two-tailed Mann–Whitney test

55 percent of the PV patients (*n* = 11) underwent a therapy switch (Table 2; PV-01 – PV-09, PV-16, and PV-18). Ten of them reached pVL below LOD after averagely 46.7 (±31.2) weeks after ART initiation and PV-18 was lost to follow up. In three of these patients, DRMs were detected (PV-03, PV-04, PV-07). Out of the group of patients without therapy switch (*n* = 9), seven patients reached suppressed pVL within 17.2 (+/- 9.2) weeks (PV-10–PV-14 and PV-19–PV-20). Comparing the average time of decline for the groups with and without therapy switch, respectively, there was no significant difference (Fig. 1). These results indicate that a switch of ART without a mutation-related indication has no beneficial impact on the outcome of PV.

### HIV-1 persistent viremia is often followed by low-level viremia

**Fig. 2:**
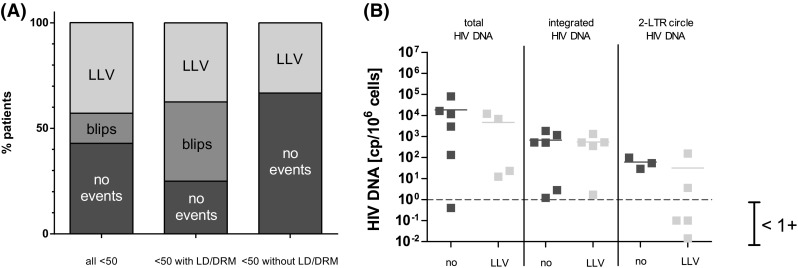
Persistent Viremia is often followed by LLV. **a** Follow-up events (no events, transient HIV-1 RNA blips, showing *up* with episodes of low-level viremia) of patients who received a first-line therapy and who reached pVL <50 RNA copies/ml were depicted (*left*). Sub-analysis of patients reaching pVL <50 who had low drug levels as well drug-resistant viruses (*middle*) and those who had not (*right*). **b** Total HIV-1 DNA, integrated proviral DNA, and non-integrated viral DNA (2-LTR circles) were determined using DNA isolated from PBMCs collected at time point of genotyping. The *lines* indicate mean values

We investigated whether PV might be associated with the occurrence of blips or of episodes of low-level viremia (LLV) during the follow-up time after the patients have reached pVL below the LOD. Here, a blip was defined as single virus detections that indicates transient viremia only, while, in contrast, LLV was defined as at least two consecutive pVL (>50 RNA copies/ml and <1000 RNA copies/ml). For this analysis, we only included patients who received a first-line therapy and who reached pVL <50 RNA copies/ml (*n* = 14; PV 01–PV14). As shown in Table 2 and Fig. 2a, we observed that 42.9% (*n* = 6/14) of these patients showed sustained suppression of HIV-RNA. However, most interestingly, we found that the percentage of patients who showed up with episodes of LLV after reaching <50 pVL was particularly high. Here, 42.9% (*n* = 6) of patients with PV showed up with low-level viremia (LLV) during the follow-up time, which, in general, was more than threefold higher tendency compared to approx. 12% in non-PV patients (see also in the “Discussion” section) [25, 26]. Subsequent blips were observed only in 21.4% (*n* = 3) of the patients.

Based on this finding, we sub-divided the group of patients finally reaching LOD (<50) regardless whether they received the first-line or multi-line therapy (*n* = 17) into two smaller groups. We compared the outcome of the group of patients having low drug levels (LD) or drug-resistant viruses (*n* = 8) with the group of patients without these features (*n* = 9) but did not found different frequencies of LLV experience after PV. Interestingly, we did not observe blips in the group without LD and DRMs, while the blip frequency in the other group was comparable to studies published previously [25, 26].

Thus, our data indicate that the risk of experiencing episodes of LLV tended to be especially high in patients who have shown up with PV.

### The probability to show up with low-level viremia after reaching the LOD is not correlated with HIV-DNA levels

We next raised the question whether the size of the viral reservoir measured as levels of HIV-DNA in PBMCs might be correlated with the occurrence of PV or LLV. Thus, we quantified non-integrated viral DNA, which can be measured by total HIV-1 DNA, integrated proviral DNA as well as 2-LTR circles derived from PBMCs that were collected during PV.

As shown in Fig. 2b, we compared the groups of PV patients experiencing LLV with the group of long-term suppressed patients with respect to the amounts of viral DNA. However, we did not find significant differences between the groups (Fig. 2b). Comparing the sub-groups of PV patients with and without drug-resistant viruses and low drug levels, respectively, we also were not able to observe a significant difference (data not shown). Thus, based on our data, we could not correlate the amounts of proviral DNA with PV and the subsequent emergence of LLV. Hence, the putative size of the viral reservoir at least at times of genotyping seems not to be a suitable marker to predict episodes of LLV that emerge after PV.

## Discussion

In this retrospective study, we analyzed 20 PV patients after the start or restart of antiretroviral treatment regimens. We found that DRMs and intermitted low drug levels could be considered as probable reasons for PV only in a sub-set of PV patients. Since the baseline pVL in this group was relatively high, we also suggest that high virus load levels might be another possible reason for a prolonged virus decay. Furthermore, the switch of ART does not per se have a beneficial impact on the outcome of PV in absence of DRMs. Most strikingly, we found that the risk of experiencing episodes of LLV tended to be especially high in patients who have shown up with PV when compared to previously published studies [25, 26]. Nevertheless, we found that the amounts of HIV-1 DNA species at least at times of genotyping were not an appropriate marker to predict episodes of LLV that emerge after PV.

Up to now, it has remained unclear whether the prolonged decline of HIV-1 pVL is related to pre-existing drug-resistant variants, the emergence of new DRMs or low drug levels. In a recent study, it has been shown that patients irrespective of therapy changes having a high pre-ART pVL (>500,000 copies/mL) show a low probability of achieving viral suppression within 72 weeks [15]. When analyzing our entire group of PV patients or sub-groups with high and low BL pVL, however, we did not find a significant correlation between pVL BL and the duration of PV, even though patients with PV tend to have high baseline viral loads (>500.000 copies/mL). Most of the patients in this cohort had pVL >100,000 copies/ml which is defined as very high. As shown in Table 1, median baseline HIV-1 RNA level (pre-ART) was 302,000 [39,473; 744,150] copies/ml. Furthermore, the median baseline (BL) CD4+ T-cell level was low 105.5 (55.5; 286.8). Thus, the combination of high pVL and low CD4+ T-cell level might be another causative reason for prolonged RNA decline in this cohort.

**Fig. 3:**
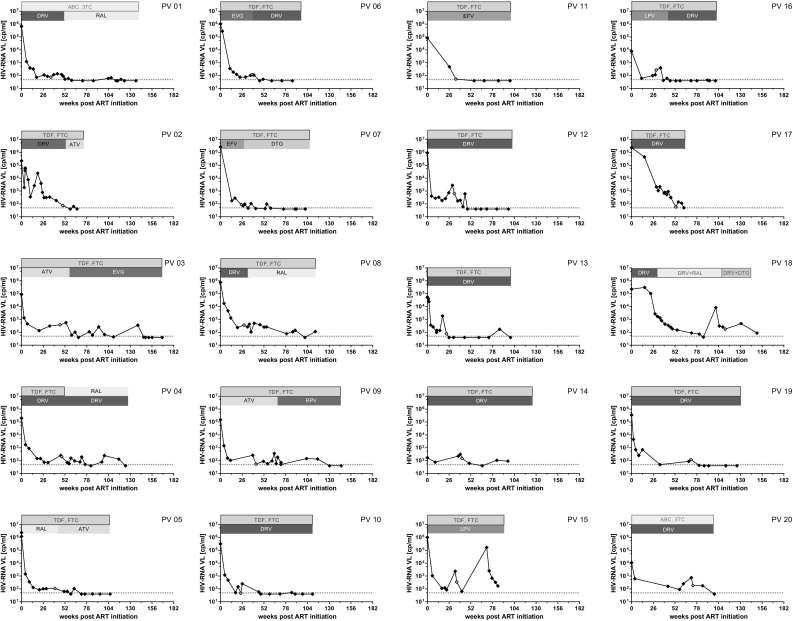
Course of viral load decline and treatment regimens. Viral load declines and treatment regimens of first-line treated patients PV-1–PV-14 and therapy-experienced patients (PV-15–PV-20) are depicted. The courses of pVL and treatment regimens are illustrated for each patient. *DRM* drug-resistance mutation, *LD* low drug level, *LF* lost to follow up before reaching pVL <50

In former studies, a significant higher risk for the development of drug resistance was detected in patients whose viral load decreased with a relatively slow rate [21]. In our study, we identified six patients (30%) carrying drug-resistant viruses; three of these were found in RNA samples. Although using NGS, we were able to identify minority variants carrying therapy-associated DRMs in two patients. Summarizing, persistent viral replication was at least in part (30% of the patients) associated with the detection of drug-resistant HIV variants. Noteworthy, NGS was shown to be a highly sensitive sequencing method allowing the identification of sub-populations at earlier time points. In addition, when compared to Sanger sequencing, significantly, more data are generated improving the quality. Hence, NGS has proven to be very helpful in clinical practice allowing an earlier adjustment of the individual ART, which might also be helpful to monitor patients with PV. Low drug levels related to poor adherence, malabsorption, insufficient dosage, drug–drug interactions, impaired intracellular metabolism, P450 hyperactivity, and P glycoprotein overexpression might have a tremendous effect on ART efficacy [13]. In our patient cohort, sub-optimal drug level was the probable reason for the prolonged RNA decline in a sub-set of patients (20%). Comparing the course of viral load decline of patients with sub-optimal drug-level (PV-02, PV-15, and PV-18), repeatedly temporarily insufficient viral suppression becomes obvious. Here, ups and downs of HIV-RNA levels without changing ART are hints for compliance issues (Fig. 3). However, the distinction between adherence disorders and resorption was not possible and specific metabolism problems of each patient could not be addressed in this study. Thus, even in patients with sufficient drug-levels in therapeutic drug monitoring (TDM), adherence disorders may have caused poor drug availability. For the remaining 50% of PV patients without drug-resistant viruses and low drug levels, it is tempting to speculate that the size of the viral reservoir was extraordinary high, which was the reason why the viral load decline took longer than usually observed in clinical practice [5], even though we were not able to observe any correlation with HIV-DNA levels obtained from PBMCs and PV. However, the initial baseline viral load, which might give a hint for the size of viral reservoir [27], was shown to influence the decline [4, 14]. In particular, Maldarelli et al. studied 145 patients whose pVL dropped below LOD within 24 weeks after ART initiation and found a significant correlation by comparing pVL at week 60 to those determined before ART initiation [14]. Furthermore, they found that both the extent of pVL reduction and the level of PV were not associated with pre-therapy CD4+ T-cell count and change in CD4+ T-cell count on therapy. In this respect, it has also been shown that HIV-1 RNA may decline more slowly, because long-lived cells are not reached by immune response like CD8^+^ cells and by antiretroviral drugs [8, 28]. Here, the loss of long-lived HIV-infected cells was shown to be a major contributor to pVL decline, whereas the activation of latently infected lymphocytes was only a minor contributor [2]. Ongoing HIV-1 replication was shown to be associated with low drug levels measured in lymphatic tissues, and hence, HIV replication can continue and make up the viral reservoir despite potent ART [8, 28]. In this respect, it has been shown that ART itself can influence the existing reservoir. In particular, boosted dual protease inhibitor regimens have a poor ability to penetrate the blood–brain barrier, which can result in residual HIV replication in the CNS [9]. Thus, there is an urgent need for novel parameters describing ongoing replication in certain compartments and tissues, i.e., sanctuary sites of replication, as well as therapeutical possibilities to intervent or improve the possibility to penetrate the replication sites.

The importance of the viral reservoir, additional host factors, or compliance issues as the underlying reason for PV is further emphasized by the fact that patients with PV in this study were especially prone to show up with low-level viremia episodes during the follow-up time. We found that the rebound probability and risk to show up with LLV after a period of PV were more than threefold higher (~43%) when compared to the above-mentioned studies by Moore et al. and Sklar et al. (~12%) [25, 26]. Here, in the cohort study of Sklar et al., 448 patients on ART on both PI-based and non-PI-based regimens with pVL copies <50 copies/ml were followed over 69 weeks. Once reached pVL <50 copies/ml, 4.2% had lasting low-level-viremia <400 copies/ml and 7.4% had lasting high-level viremia >400 copies/ml, which was 11.6% in total [26]. In addition, 27.2% had transient viremia only. In the study of Moore et al., 553 patients on ART (73% on PI regimen) with pVL copies <50 copies/ml were followed over 56 weeks. Here, patients were analyzed being under the limit of detection up to 120 weeks. In average, analyzing these patients 35% had blips, while 12.8% had at least two RNA measurements >50 copies/ml. In our study, blips were observed more frequently in the group of patients having low drug levels and/or drug-resistant viruses when compared with those patients who did not. Blips are frequently seen even in patients who are effectively suppressed for years. This might be explained due to immune activation related to, for instance, respiratory infections or vaccination but are not correlated with treatment failure [29, 30]. However, blips might at least in a sub-set of patients also be associated with compliance issues. Importantly, the detection of consecutive pVL is suspicious for ongoing viral replication [31]. It has been also shown that LLV may be predictive for viral rebound and that resistance mutations emerging during episodes of low-level viremia might lead to virological failure [32–35].

Since the overall number of patients in our study was low and multiple factors seemed to independently influence the occurrence of PV, this study has certain limitations. In addition, the patients studied were treated with different ART regimens, so the influence of drugs on PV could not be adequately evaluated. In a comparative analysis of treatment-naïve patients receiving NRTI and INSTI versus NNRTI and boosted PI, for instance, the benefit of INSTI, in particular raltegravir, on RNA plasma decline comes obvious [36]. Since the patients, in our study, have been treated in the time period from 2012 till 2015, a high frequency of PI-based ART can be observed. Only two patients received NRTIs + INSTI as first-line therapy and six patients switched their ART to an INSTI-containing regimen. A comparison of these unequal groups was, therefore, not possible; however, it will be interesting to investigate newly acquired data on this comparison in future studies. Since it was not possible to uniformly collect drug level data in this study population, we compared *C*_min_ values of each drug and therapy application form to determine whether drug levels were available in sufficient quantities. The drug levels were measured only once at the time of genotyping, so that only a strong generalized representation is possible. Although our sample size was small, this work clearly showed the heterogeneity of the underlying factors of PV and the urgent need of identifying markers for classifying PV according to its clinical outcome. The major finding of this study, however, was the observation that PV appears to be a risk factor for undergo episodes of LLV.
